# The HCV care continuum among people who use drugs: protocol for a systematic review and meta-analysis

**DOI:** 10.1186/s13643-016-0293-6

**Published:** 2016-07-11

**Authors:** Jennifer R. Reed, Ashly E. Jordan, David C. Perlman, Daniel J. Smith, Holly Hagan

**Affiliations:** College of Nursing, New York University, 422 First Avenue, New York, NY 10010 USA; Center for Drug Use and HIV Research, New York, NY USA; Mount Sinai Beth Israel, New York, NY USA

**Keywords:** People who use drugs, Hepatitis c, Hepatitis c care continuum, Healthcare access, Systematic review, Meta-analysis

## Abstract

**Introduction:**

The diagnosis, management, and treatment for hepatitis C virus (HCV) infection (the “HCV care continuum”) have improved in recent years. People who use drugs (PWUD) have a prevalence of HCV infection from 30 to 70 %, yet rates of testing, engagement in care, and treatment for HCV are disproportionately low compared to other populations. Delineating the progression of PWUD through the steps in the HCV care continuum in the USA is important in informing efforts to improve HCV outcomes among PWUD.

**Methods/design:**

Scientific databases will be searched using a comprehensive automated search strategy; gray literature and reference lists will be manually searched. Eligible reports will provide original research data related to the HCV care continuum in the USA including proportions of PWUD engaging in the following discrete steps: screening/testing, engagement in care (including receiving an HCV clinical assessment), treatment initiation and completion, and rates of those with successful HCV treatment. A quality-rating tool will be developed to ascertain the level of bias (including selection bias) in each report, and a quality score will be assigned to each eligible report. A tool adapted from the Pragmatic Explanatory Continuum Indicator Summary-2 instrument will be developed to assess the extent to which an included report reflects an effectiveness or efficacy study design. Pooled estimates and measures of association will be calculated using random effects models, and heterogeneity will be assessed at each stage of data synthesis.

**Discussion:**

Through this review, we hope to quantify the proportion of PWUD at each progressive step and to help identify key individual, social, and structural points of leakage in the HCV care continuum for PWUD. In meeting these objectives, we will identify predictors to progress along the HCV care continuum, which can be used to inform policy to directly improve HCV care for PWUD.

**Systematic review registration:**

PROSPERO CRD42016034113

**Electronic supplementary material:**

The online version of this article (doi:10.1186/s13643-016-0293-6) contains supplementary material, which is available to authorized users.

## Introduction

Hepatitis C virus (HCV) is a chronic, life-threatening blood-borne infection that affects an estimated 150 million people globally [1]. It is most efficiently transmitted by percutaneous exposure, which makes people who inject drugs (PWID) at particularly high risk [2, 3]. In the USA, there are an estimated 1.86 million PWID, of whom 1.5 million (74 %) are believed to be HCV antibody positive [4]. People who use drugs (PWUD) administered via smoking or inhalation are also at risk as a result of unprotected sex and sharing pipes or straws that may be blood contaminated [5, 6]. Additionally, there have been outbreaks of HCV among prescription opioid users who have recently transitioned to drug injection, as well as among HIV-positive men who have sex with men [7–12]. PWUD are often infected with HCV at an early age and thus are at risk for developing liver fibrosis, cirrhosis, and hepatocellular carcinoma in mid-adulthood, potentially resulting in morbidity and mortality among individuals in their most productive period of life [13–16]. Projections suggest that increases in the incidence of HCV-related cirrhosis and its complications will continue for at least two to three decades [16]. Among HCV mono-infected individuals, an estimated 20–25 % will develop liver disease, which may manifest as fibrosis, cirrhosis, end-stage liver disease, or hepatocellular carcinoma [15]. HIV co-infection accelerates this progression [17, 18].

HCV treatment has the potential to significantly reduce morbidity and mortality associated with chronic HCV infection [19, 20]. There have been dramatic advances in the ability to treat chronic HCV infection; more efficacious and tolerable medications requiring shorter treatment courses can lead to a sustained virologic response (SVR), which predicts reduced liver disease morbidity and mortality in a majority of treated patients. In addition to improvements with treatment, there have been improvements in screening methods and in diagnosis [19–24].

However, among PWUD, there are very significant gaps in the HCV care continuum that reduce the real-world population-level effectiveness of HCV treatment [25–28]. Among PWUD with HCV infection, many have not been screened or, if screened, are unaware they are infected; they may be tested for HCV antibody (a measure of the previous exposure but not necessarily the current active infection) but not for viral load to confirm active infection, and the majority are not evaluated for treatment or offered treatment [28–31]. Following treatment and cure, they may also be at risk of re-infection due to continuous exposure to HCV infection through persistent risk behaviors [32, 33].

This systematic review and meta-analysis (SR/MA) will focus on the HCV care continuum for PWUD. The HCV care continuum will be examined considering the following steps: initial screening for HCV antibodies, confirmatory diagnosis with HCV RNA testing, and provider evaluation for treatment; offer, acceptance, and initiation of treatment; adherence to treatment; completion of treatment; and achievement of SVR. This SR/MA will also examine rates of re-infection among those who achieved SVR.

### Methods

This protocol was developed in accordance with the Preferred Reporting Items for Systematic Review and Meta-Analysis Protocols (PRISMA-P) guidelines [34]. A populated PRISMA-P checklist is provided for this protocol as Additional file 1.

#### Exposure measures

The primary exposures examined in this review include (1) being a current PWUD or PWID and therefore being at risk for HCV infection and (2) being a current or former PWUD or PWID and having HCV infection. Chronic HCV infection is defined as the presence of HCV viremia. The term “PWUD” will be used to refer those who use or have used any illicit drug or drugs by any route, but will exclude those who use, or have used, alcohol, tobacco, or marijuana only. The term PWID will be used to refer to those who use or have used any illicit drug by injection.

#### Inclusion and exclusion criteria

This study will include published and unpublished data from both observational (prospective and retrospective cohort, case-control, and cross-sectional) and experimental (randomized controlled trials, hybrid designs, and quasi-experimental trials) study designs. Reports published in English from January 1, 1990, through February 20, 2016, will be included if they report on the rates of transition of the past or current PWUD or PWID from at least one step of the care continuum to another. PWUD with either HCV mono-infection or co-infection with HCV/HIV will be included in this review. Reports must include ten or more subjects who are former or current PWUD. If data is presented for both those who do and do not have a history of drug use, the report must provide disaggregated data on PWUD or PWID.

For the purposes of this SR/MA, drug use will be defined as the use of any drug, either licit or illicit, with the exception of the exclusive use of alcohol, tobacco, or marijuana. Four populations that will be specifically examined include (1) people who currently inject drugs, (2) people who formerly injected drugs, (3) people who currently use drugs without injecting, and (4) people who formerly used drugs without injecting. Reports will be eligible if they examine people in one or more of these four groups as they are identified as having completed one or more steps on the HCV care continuum.

Inclusion criteria will require that reports present data from the USA, as the progression through steps in the care continuum is highly dependent on the type of healthcare delivery system in a given country. Thus, we will restrict our initial analysis for this SR/MA to the progression of PWUD through the HCV care continuum in the USA. However, we will retain the list of non-US studies that are otherwise eligible for inclusion for possible analysis at a later date.

#### Outcome measures

Care continuum steps to be considered will include (a) HCV antibody screening (where testing only confirms exposure to the HCV virus and not HCV viremia) and subsequent confirmatory HCV viral load testing (where HCV viremia or chronic HCV infection is ascertained); (b) linkages to next steps of HCV clinical evaluation, including evaluation for appropriate HCV treatment and offer of HCV pharmacological treatment (evaluation for HCV treatment may include liver biopsy, liver biomarkers (e.g., FIB-4), and non-invasive transient liver elastography), and offer of HCV treatment involves either a verbal recommendation for treatment by a provider or receipt of a written prescription; (c) interventions to increase patient engagement, which includes acceptance of treatment, initiation of treatment, and adherence to treatment (acceptance of treatment is willingness to begin treatment for HCV; initiation of treatment refers to taking one or more prescribed HCV medication at least once; and adherence refers to taking a specified proportion of prescribed HCV medication for a specified time, as defined by the studies reviewed); (d) completion of treatment refers to remaining in treatment until finishing the entire length of the prescribed treatment regimen; (e) achieving SVR is defined as having undetectable HCV RNA 24 weeks after completion of treatment (or at a specified time post-treatment as defined by the studies included in the review); and (f) re-infection is defined as those who have a positive HCV RNA test with a different HCV genotype or strain within 1 year of having previously achieving SVR. Each successive step in the HCV care continuum is dependent on passing through the previous step.

Each step in the HCV care continuum is contingent upon completion of the previous step. Therefore, each outcome measure listed previously is also a measure of exposure, with the exception of the last step (re-infection). We will present data for PWUD as a whole and for the subset of PWUD who are PWID where such data are available so that the proportions of PWUD attribute to PWID are made explicit.

### Search strategy

After consultation with a medical librarian, a search strategy will be conducted of PubMed, Embase, the Cumulative Index of Nursing and Allied Health Literature, and PsycInfo. A preliminary search strategy for each of these four databases is provided in Additional file 2. Keywords and MeSH terms will be included in the search if they relate to hepatitis C infection, past or current drug use, and any step along the care continuum. Ancestry searches will be conducted of reviews found in the search for additional relevant literature, and unpublished literature will be obtained by searching conference abstracts and contacting authors where appropriate. Conference abstracts to be searched include the College on Problems of Drug Dependence (CPDD), the American Association for the Study of Liver Diseases (AASLD), the European Association for the Study of the Liver (EASL), the International Harm Reduction Conference (IHRC), and the Conference on Retroviruses and Opportunistic Infections (CROI).

### Screening and data collection

Reports obtained from the search strategy will be imported into EndNote X7.5 and duplicates will be removed. The project director (PD; AEJ) and research assistant (RA; JR) will screen each abstract retrieved from the automated searches and discard those abstracts that are clearly ineligible. Full-text articles that are deemed as potentially eligible will be retrieved, and the report will be evaluated by the PD and RA to determine whether the article meets the inclusion criteria. Reasons for exclusion during full-text review will be recorded and presented as per the PRISMA guidelines. The principal investigator (PI; HH), co-investigator (Co-I; DCP), and PD will screen all articles that were unclear as to whether or not they meet the inclusion criteria on the initial review.

A final set of eligible articles will be compiled for coding. The coding will be carried out by the PD and RA. The content and structure of the coding form will be tailored to collect data on the numbers and proportions of PWUD completing each step of the HCV care continuum. The coding form will include study design elements and sample characteristics (e.g., demographic information) and will be designed to be flexible in order to accommodate varying degrees of complexity in the data.

### Quality assessment

#### Overview

Screening and data abstraction will be conducted by the PD and RA, both of whom have graduate-level training in the research methodology of systematic reviews and meta-analyses, as well as additional training in the epidemiology of HCV and drug use. Periodic staff meetings will be conducted to resolve any questions or inconsistencies in the extraction and analysis of the data. A study guide will be created to guide the process and record special cases and their resolution.

#### Study quality and critical appraisal

The quality of each included report will be assessed using a quality-rating tool adapted from the assessment of risk of bias studies developed by Hayden et al. [35]. The quality-rating tool will assess threats to validity, including selection bias, non-comparability, and misclassification.

#### Categorization of study design

Reports will be evaluated with respect to the degree to which they have features reflecting either efficacy or effectiveness study design elements, using an adaptation of the Pragmatic Explanatory Continuum Indicator Summary-2 (PRECIS-2) instrument [36, 37]. PRECIS-2 is designed to assist in the designing of studies [37]; we will adapt the instrument to facilitate post hoc evaluation. Studies are typically not purely efficacy studies or effectiveness studies but instead fall somewhere on the efficacy/effectiveness continuum. The adapted PRECIS-2 tool will allow us to evaluate where the reports included in this review fall on this continuum.

#### Selection bias

In case-control studies, selection bias will be evaluated by examining whether adequate methods were used to classify cases and controls and whether cases and controls came from the same underlying population. In cohort studies, selection bias will be assessed to determine if selection of the cohort was related to the likelihood of their progression through the care continuum.

#### Comparability

Comparability of cases and controls will be assessed by ensuring that methods were used to adjust for confounding between the two groups. In cohort studies, comparability will be assessed by ensuring that the association between the exposure and the outcome adjusted for important differences between the exposed and unexposed cohorts.

#### Misclassification

It is important that the studies included in this review provide an explicit definition of each exposure and outcome in order to avoid misclassification. In addition, misclassification will be addressed as part of eligibility screening; for example, inclusion criteria will require that HCV infection be confirmed via serology testing to avoid misclassification of exposure.

### Data analysis

Beginning February 2016, we will conduct database searches using appropriate keywords and download all relevant titles to EndNote version X7.5 for data management.

The analyses will define each step in the continuum discretely for each report, from testing to SVR, and aggregate the study-level data for synthesis. Synthesis will begin with the search for homogeneous subsets within sets of studies, followed by meta-analysis and calculation of summary estimates within the homogeneous subsets. Graphical and statistical analysis will be conducted using software designed specifically for meta-analysis.

If the systematic review results in a small number of studies for the meta-analysis, the estimates of heterogeneity among the included studies may be inappropriately low (as an artifact of the small number of studies) [38]. We will be wary of this and will not assume homogeneity in the presence of low heterogeneity, and we will not conduct simple fixed-effects meta-analysis; meta-analysis and random effects meta-regression will be carried out instead.

Variability in effects among the studies may reflect important differences or confounding by other factors. Therefore, the evidence of heterogeneity will be evaluated at each step in the analysis to distinguish between true variation of effects and heterogeneity due to other differences. We will report both *I*^2^ and *H*^2^ with confidence intervals as measures of heterogeneity, since *I*^2^ alone is not sufficient when conducting meta-analyses on a small number of studies [39]. Subgroup analyses of those with or without HIV co-infection will be assessed as well after review of the included studies to determine if differences exist in the HCV care continuum between these two groups.

Publication bias will be assessed if the final analysis consists of at least ten studies, since this is the minimum number required to achieve adequate power [40]. This will be accomplished by comparing estimates between published and unpublished reports and by the use of funnel plots.

## Discussion

This systematic review and meta-analysis will critically assess the progression of PWUD through the steps in the HCV care continuum. We anticipate the benefits of this review to be twofold: first, we anticipate that this review will quantify the proportion of PWUD at each progressive step in the care continuum and, thus, present a robust characterization of the engagement of PWUD in the sequential steps of the HCV care continuum that can be used in modeling and public health planning efforts. Second, we hope that this review will help identify key individual, social, and structural bottlenecks or points of leakage in the care continuum. In addition to identifying the proportions of PWUD/PWID progressing through care continuum steps, we will examine studies for the presence of covariates which, if reported and present, may be associated with progression through care continuum steps in an attempt to identify barriers to and facilitators of progression. This will help identify points where interventions are needed to improve individual and population-level progress through the care continuum and will help to elucidate modifiable barriers, facilitators, and predictors of progression through the care continuum, including those relevant at each step and those relevant to achieving SVR via HCV treatment. Additionally, the review’s focus on providing HCV re-infection rates post-SVR among PWUD has the potential to inform interventions on secondary prevention of re-infection among high-risk individuals.

## Abbreviations

HCV, hepatitis C virus; PRECIS-2, Pragmatic Explanatory Continuum Indicator Summary-2; PRISMA-P, Preferred Reporting Items for Systematic Review and Meta-Analysis Protocols; PWID, people who inject drugs; PWUD, people who use drugs; SVR, sustained virologic response

## Additional files

Additional file 1: PRISMA-P 2015 Checklist. Additional file 2: Search strategy for CINAHL (via EBSCO), Embase (via Ovid), PsycInfo (via Ovid), and PubMed (via Medline).

## References

[1] Averhoff FM, Glass N, Holtzman D (2012). Global burden of hepatitis C: considerations for healthcare providers in the United States. Clin Infect Dis.

[2] Mehta SH, Astemborski J, Kirk GD (2011). Changes in blood-borne infection risk among injection drug users. J Infect Dis.

[3] Nelson PK, Mathers BM, Cowie B (2011). Global epidemiology of hepatitis B and hepatitis C in people who inject drugs: results of systematic reviews. Lancet.

[4] Friedman SR, Tempalski B, Cooper H (2004). Estimating numbers of injecting drug users in metropolitan areas for structural analyses of community vulnerability and for assessing relative degrees of service provision for injecting drug users. J Urban Health.

[5] Scheinmann R, Hagan H, Lelutiu-Weinberger C (2007). Non-injection drug use and hepatitis C virus: a systematic review. Drug Alcohol Depend.

[6] Howe CJ, Fuller CM, Ompad DC (2005). Association of sex, hygiene and drug equipment sharing with hepatitis C virus infection among non-injecting drug users in New York City. Drug Alcohol Depend.

[7] Cropsey KL, Lane PS, Perkins AC (2013). Buprenorphine and medication management in a community corrections population: a pilot study. J Addict Med.

[8] Hagan H, Jordan AE, Neurer J, Cleland CM (2015). Incidence of sexually transmitted hepatitis C virus infection in HIV-positive MSM: a systematic review and meta-analysis. AIDS.

[9] Jordan AE, Perlman DC, Neurer J, Smith DJ, Des Jarlais DC, Hagan H. Prevalence of hepatitis C virus infection among HIV+ men who have sex with men: a systematic review and meta-analysis. Int J STD AIDS. 2016; [Epub ahead of print]. 10.1177/0956462416630910 PMC4965334 26826159

[10] Fierer DS, Uriel AJ, Carriero DC (2009). Characterization of an epidemic of sexually-transmitted acute hepatitis C infection in HIV-infected men in New York City. Hepatology.

[11] van de Laar TJ, van der Bij AK, Prins M (2007). Increase in HCV incidence among men who have sex with men in Amsterdam most likely caused by sexual transmission. J Infect Dis.

[12] van der Helm JJ, Prins M, del Amo J (2011). The hepatitis C epidemic among HIV-positive MSM: incidence estimates from 1990 to 2007. AIDS.

[13] Hagan H, Des Jarlais DC, Stern R (2007). HCV synthesis project: preliminary analyses of HCV prevalence in relation to age and duration of injection. Int J Drug Policy.

[14] Smith DJ, Combellick J, Jordan AE, Hagan H (2015). Hepatitis C virus (HCV) disease progression in people who inject drugs (PWID): a systematic review and meta-analysis. Int J Drug Policy.

[15] Thomas DL, Astemborski J, Rai RM (2000). The natural history of hepatitis C virus infection: host, viral, and environmental factors. JAMA.

[16] Wong JB, McQuillan GM, McHutchison JG, Poynard T (2000). Estimating future hepatitis C morbidity, mortality, and costs in the United States. Am J Public Health.

[17] Brau N (2003). Update on chronic hepatitis C in HIV/HCV-coinfected patients: viral interactions and therapy. AIDS.

[18] Operskalski EA, Kovacs A (2011). HIV/HCV co-infection: pathogenesis, clinical complications, treatment, and new therapeutic technologies. Curr HIV/AIDS Rep.

[19] Sulkowski MS, Gardiner DF, Rodriguez-Torres M (2014). Daclatasvir plus sofosbuvir for previously treated or untreated chronic HCV infection. N Engl J Med.

[20] van der Meer AJ, Veldt BJ, Feld JJ (2012). Association between sustained virological response and all-cause mortality among patients with chronic hepatitis C and advanced hepatic fibrosis. JAMA.

[21] Dhaliwal HS, Nampoothiri RV (2014). Daclatasvir plus sofosbuvir for HCV infection. N Engl J Med.

[22] Younossi ZM, Stepanova M, Nader F, et al. Patient-reported outcomes in chronic hepatitis C patients with cirrhosis treated with sofosbuvir-containing regimens. Hepatology. 2014;59(6):2161–9. 10.1002/hep.27161 24710669

[23] Poordad F, Hezode C, Trinh R (2014). ABT-450/r-ombitasvir and dasabuvir with ribavirin for hepatitis C with cirrhosis. N Engl J Med.

[24] Afdhal N, Reddy KR, Nelson DR (2014). Ledipasvir and sofosbuvir for previously treated HCV genotype 1 infection. N Engl J Med.

[25] Al-Tayyib AA, Thiede H, Burt RD, Koester S (2015). Unmet health care needs and hepatitis C infection among persons who inject drugs in Denver and Seattle, 2009. Prev Sci.

[26] Harris M, Rhodes T (2013). Hepatitis C treatment access and uptake for people who inject drugs: a review mapping the role of social factors. Harm Reduct J.

[27] Meyer JP, Moghimi Y, Marcus R, Lim JK, Litwin AH, Altice FL. Evidence-based interventions to enhance assessment, treatment, and adherence in the chronic hepatitis C care continuum. Int J Drug Policy. 2015; 26(10):922–35. 10.1016/j.drugpo.2015.05.002 PMC4577454 26077144

[28] Linas BP, Barter DM, Leff JA (2014). The hepatitis C cascade of care: identifying priorities to improve clinical outcomes. PLoS One.

[29] Aspinall EJ, Doyle JS, Corson S (2015). Targeted hepatitis C antibody testing interventions: a systematic review and meta-analysis. Eur J Epidemiol.

[30] Jordan AE, Masson CL, Mateu-Gelabert P (2013). Perceptions of drug users regarding hepatitis C screening and care: a qualitative study. Harm Reduct J.

[31] Mehta SH, Lucas GM, Mirel LB (2006). Limited effectiveness of antiviral treatment for hepatitis C in an urban HIV clinic. AIDS.

[32] Grebely J, Prins M, Hellard M, et al. Hepatitis C virus clearance, reinfection, and persistence, with insights from studies of injecting drug users: towards a vaccine. Lancet Infect Dis. 2012 2014-03-21 2012;12(5):408-414. 10.1016/S1473-3099(12)70010-5 PMC3608418 22541630

[33] Page K, Hahn JA, Evans J (2009). Acute hepatitis C virus infection in young adult injection drug users: a prospective study of incident infection, resolution, and reinfection. J Infect Dis.

[34] Moher D, Shamseer L, Clarke M (2015). Preferred reporting items for systematic review and meta-analysis protocols (PRISMA-P) 2015 statement. Syst Rev.

[35] Hayden JA, Côté P, Bombardier C. Evaluation of the quality of prognosis studies in systematic reviews. Ann Intern Med. 2006 Mar 21 2013-04-25 2006;144(6):427-437. 10.7326/0003-4819-144-6-200603210-00010 16549855

[36] Thorpe KE, Zwarenstein M, Oxman AD (2009). A pragmatic-explanatory continuum indicator summary (PRECIS): a tool to help trial designers. CMAJ.

[37] Loudon K, Treweek S, Sullivan F, Donnan P, Thorpe KE, Zwarenstein M (2015). The PRECIS-2 tool: designing trials that are fit for purpose. BMJ.

[38] Kontopantelis E, Springate DA, Reeves D (2013). A re-analysis of the Cochrane Library data: the dangers of unobserved heterogeneity in meta-analyses. PLoS One.

[39] Mittlbock M, Heinzl H (2006). A simulation study comparing properties of heterogeneity measures in meta-analyses. Stat Med.

[40] Sterne JA, Gavaghan D, Egger M (2000). Publication and related bias in meta-analysis: power of statistical tests and prevalence in the literature. J Clin Epidemiol.

