# Supplementary material for: The HCV care continuum among people who use drugs: protocol for a systematic review and meta-analysis
Source: Syst Rev. 2016 Jul 11;5:110. doi: 10.1186/s13643-016-0293-6 (PMC4940695; doi:10.1186/s13643-016-0293-6)
Supplement: Additional file 2: — Search strategy for CINAHL (via EBSCO), Embase (via Ovid), PsycInfo (via Ovid), and PubMed (via Medline). [file 13643_2016_293_MOESM2_ESM.docx]

**Additional File 2**

**Search Strategy for CINAHL (via EBSCO), Embase (via Ovid), PsycInfo (via Ovid), and PubMed (via Medline)**

**CINAHL (via EBSCO)**

*1/1/1990-2/20/2016*

| S75 | S23 AND S74 |
| --- | --- |
| S74 | S33 OR S43 OR S57 OR S61 OR S66 OR S73 |
| S73 | S67 OR S68 OR S69 OR S70 OR S71 OR S72 |
| S72 | "coordination of care" |
| S71 | "care coordination" |
| S70 | "model of care" |
| S69 | "care cascade" |
| S68 | "continuum of care" |
| S67 | "care continuum" |
| S66 | S62 OR S63 OR S64 OR S65 |
| S65 | "re-infection" |
| S64 | "reinfection" |
| S63 | "recurrence" |
| S62 | (MH "Recurrence") |
| S61 | S58 OR S59 OR S60 |
| S60 | "SVR" |
| S59 | "sustained virologic response" |
| S58 | (MH "Treatment Outcomes+") |
| S57 | S44 OR S45 OR S46 OR S47 OR S48 OR S49 OR S50 OR S51 OR S52 OR S53 OR S54 OR S55 OR S56 |
| S56 | "treatment retention" |
| S55 | "retention in treatment" |
| S54 | "care retention" |
| S53 | "retention in care" |
| S52 | "treatment engagement" |
| S51 | "care engagement" |
| S50 | "engagement in care" |
| S49 | "treatment completion" |
| S48 | "treatment adherence" |
| S47 | "treatment initiation" |
| S46 | "treatment uptake" |
| S45 | "treatment willingness" |
| S44 | (MH "Patient Compliance+") |
| S43 | S34 OR S35 OR S36 OR S37 OR S38 OR S39 OR S40 OR S41 OR S42 |
| S42 | HCV n10 evaluation |
| S41 | HCV n10 management |
| S40 | HCV n10 "linkage to care" |
| S39 | HCV n10 referral |
| S38 | HCV n10 assessment |
| S37 | (MH "Referral and Consultation+") |
| S36 | (MH "Patient Assessment+") |
| S35 | (MH "Health Care Delivery+") |
| S34 | (MH "Continuity of Patient Care+") |
| S33 | S24 OR S25 OR S26 OR S27 OR S28 OR S29 OR S30 OR S31 OR S32 |
| S32 | HCV n10 "viral load" |
| S31 | HCV n10 antibodies |
| S30 | HCV n10 antibody |
| S29 | HCV n10 testing |
| S28 | HCV n10 screening |
| S27 | (MH "Antibodies+") |
| S26 | (MH "Diagnostic Tests, Routine") |
| S25 | (MH "Point-of-Care Testing") |
| S24 | (MH "Health Screening+") |
| S23 | S16 AND S22 |
| S22 | S17 OR S18 OR S19 OR S20 OR S21 |
| S21 | "hepatitis non a non b" |
| S20 | "HCV" |
| S19 | "hep c" |
| S18 | "hepatitis c" |
| S17 | (MH "Hepatitis C+") |
| S16 | S1 OR S2 OR S3 OR S4 OR S5 OR S6 OR S7 OR S8 OR S9 OR S10 OR S11 OR S12 OR S13 OR S14 OR S15 |
| S15 | "drug dependence" |
| S14 | "drug abuse" |
| S13 | "drug use" |
| S12 | "substance dependence" |
| S11 | "substance abuse" |
| S10 | "substance use" |
| S9 | (MH "Substance Dependence+") |
| S8 | (MH "Substance Abuse+") |
| S7 | "IVDU" |
| S6 | "IDU" |
| S5 | "PWID" |
| S4 | "persons who inject drugs" |
| S3 | "people who inject drugs" |
| S2 | (MH "Intravenous Drug Users") |
| S1 | (MH "Substance Abuse, Intravenous") |

**Embase (via Ovid)**

*1/1/1990-2/20/2016*

| 1. | exp screening/ |
| --- | --- |
| 2. | exp "point of care testing"/ |
| 3. | exp viral load testing/ |
| 4. | exp dried blood spot testing/ |
| 5. | exp hepatitis C antibody/ |
| 6. | exp hepatitis C rapid test/ |
| 7. | (HCV adj10 screening).ti,ab,kw. |
| 8. | (HCV adj10 testing).ti,ab,kw. |
| 9. | (HCV adj10 antibody).ti,ab,kw. |
| 10. | (HCV adj10 antibodies).ti,ab,kw. |
| 11. | (HCV adj10 "viral load").ti,ab,kw. |
| 12. | 1 or 2 or 3 or 4 or 5 or 6 or 7 or 8 or 9 or 10 or 11 |
| 13. | exp patient care/ |
| 14. | exp health care delivery/ |
| 15. | exp health care access/ |
| 16. | (HCV adj10 assessment).ti,ab,kw. |
| 17. | (HCV adj10 referral).ti,ab,kw. |
| 18. | (HCV adj10 "linkage to care").ti,ab,kw. |
| 19. | 13 or 14 or 15 or 16 or 17 or 18 |
| 20. | exp patient attitude/ |
| 21. | "treatment willingness".ti,ab,kw. |
| 22. | "treatment uptake".ti,ab,kw. |
| 23. | "treatment initiation".ti,ab,kw. |
| 24. | "treatment adherence".ti,ab,kw. |
| 25. | "treatment completion".ti,ab,kw. |
| 26. | "engagement in care".ti,ab,kw. |
| 27. | "care engagement".ti,ab,kw. |
| 28. | "treatment engagement".ti,ab,kw. |
| 29. | "retention in care".ti,ab,kw. |
| 30. | "care retention".ti,ab,kw. |
| 31. | "retention in treatment".ti,ab,kw. |
| 32. | "treatment retention".ti,ab,kw. |
| 33. | 20 or 21 or 22 or 23 or 24 or 25 or 26 or 27 or 28 or 29 or 30 or 31 or 32 |
| 34. | exp treatment outcome/ |
| 35. | "sustained virologic response".ti,ab,kw. |
| 36. | "SVR".ti,ab,kw. |
| 37. | 34 or 35 or 36 |
| 38. | exp recurrent disease/ |
| 39. | "recurrence".ti,ab,kw. |
| 40. | "reinfection".ti,ab,kw. |
| 41. | "re-infection".ti,ab,kw. |
| 42. | 38 or 39 or 40 or 41 |
| 43. | "care continnum".ti,ab,kw. |
| 44. | "continuum of care".ti,ab,kw. |
| 45. | "care cascade".ti,ab,kw. |
| 46. | "model of care".ti,ab,kw. |
| 47. | "care coordination".ti,ab,kw. |
| 48. | "coordination of care".ti,ab,kw. |
| 49. | 43 or 44 or 45 or 46 or 47 or 48 |
| 50. | exp intravenous drug abuse/ |
| 51. | "people who inject drugs".ti,ab,kw. |
| 52. | "persons who inject drugs".ti,ab,kw. |
| 53. | PWID.ti,ab,kw. |
| 54. | IDU.ti,ab,kw. |
| 55. | IVDU.ti,ab,kw. |
| 56. | exp addiction/ |
| 57. | exp drug abuse/ |
| 58. | "substance use".ti,ab,kw. |
| 59. | "substance abuse".ti,ab,kw. |
| 60. | "substance dependence".ti,ab,kw. |
| 61. | "drug use".ti,ab,kw. |
| 62. | "drug abuse".ti,ab,kw. |
| 63. | "drug dependence".ti,ab,kw. |
| 64. | 50 or 51 or 52 or 53 or 54 or 55 or 56 or 57 or 58 or 59 or 60 or 61 or 62 or 63 |
| 65. | exp hepatitis C/ |
| 66. | exp Hepatitis C virus/ |
| 67. | "hepatitis c".af. |
| 68. | "hep c".af. |
| 69. | "HCV".af. |
| 70. | "hepatitis non a non b".af. |
| 71. | 65 or 66 or 67 or 68 or 69 or 70 |
| 72. | 64 and 71 |
| 73. | 12 or 19 or 33 or 37 or 42 or 49 |
| 74. | 72 and 73 |

**PsycInfo (via Ovid)**

1/1/1990-2/20/2016

| 1. | exp intravenous drug usage/ |
| --- | --- |
| 2. | "people who inject drugs".ti,ab. |
| 3. | "persons who inject drugs".ti,ab. |
| 4. | PWID.ti,ab. |
| 5. | IDU.ti,ab. |
| 6. | IVDU.ti,ab. |
| 7. | exp addiction/ |
| 8. | exp drug abuse/ |
| 9. | "substance use".ti,ab. |
| 10. | "substance abuse".ti,ab. |
| 11. | "substance dependence".ti,ab. |
| 12. | "drug use".ti,ab. |
| 13. | "drug abuse".ti,ab. |
| 14. | "drug dependence".ti,ab. |
| 15. | 1 or 2 or 3 or 4 or 5 or 6 or 7 or 8 or 9 or 10 or 11 or 12 or 13 or 14 |
| 16. | exp Hepatitis/ |
| 17. | "hepatitis c".af. |
| 18. | "hep c".af. |
| 19. | "HCV".af. |
| 20. | "hepatitis non a non b".af. |
| 21. | 16 or 17 or 18 or 19 or 20 |
| 22. | 15 and 21 |
| 23. | exp screening/ |
| 24. | exp antibodies/ |
| 25. | exp testing/ |
| 26. | (HCV adj10 screening).ti,ab. |
| 27. | (HCV adj10 testing).ti,ab. |
| 28. | (HCV adj10 antibody).ti,ab. |
| 29. | (HCV adj10 antibodies).ti,ab. |
| 30. | (HCV adj10 "viral load").ti,ab. |
| 31. | 23 or 24 or 25 or 26 or 27 or 28 or 29 or 30 |
| 32. | exp Health Care Delivery/ |
| 33. | (HCV adj10 assessment).ti,ab. |
| 34. | (HCV adj10 referral).ti,ab. |
| 35. | (HCV adj10 "linkage to care").ti,ab. |
| 36. | 32 or 33 or 34 or 35 |
| 37. | exp client attitudes/ |
| 38. | exp Treatment Barriers/ |
| 39. | exp treatment compliance/ |
| 40. | "treatment willingness".ti,ab. |
| 41. | "treatment uptake".ti,ab. |
| 42. | "treatment initiation".ti,ab. |
| 43. | "treatment adherence".ti,ab. |
| 44. | "treatment completion".ti,ab. |
| 45. | "engagement in care".ti,ab. |
| 46. | "care engagement".ti,ab. |
| 47. | "treatment engagement".ti,ab. |
| 48. | "retention in care".ti,ab. |
| 49. | "care retention".ti,ab. |
| 50. | "retention in treatment".ti,ab. |
| 51. | "treatment retention".ti,ab. |
| 52. | 37 or 38 or 39 or 40 or 41 or 42 or 43 or 44 or 45 or 46 or 47 or 48 or 49 or 50 or 51 |
| 53. | exp Treatment Outcomes/ |
| 54. | "sustained virologic response".ti,ab. |
| 55. | "SVR".ti,ab. |
| 56. | 53 or 54 or 55 |
| 57. | "recurrence".ti,ab. |
| 58. | "reinfection".ti,ab. |
| 59. | "re-infection".ti,ab. |
| 60. | 57 or 58 or 59 |
| 61. | exp "continuum of care"/ |
| 62. | "care continuum".ti,ab. |
| 63. | "continuum of care".ti,ab. |
| 64. | "care cascade".ti,ab. |
| 65. | "model of care".ti,ab. |
| 66. | "care coordination".ti,ab. |
| 67. | "coordination of care".ti,ab. |
| 68. | 61 or 62 or 63 or 64 or 65 or 66 or 67 |
| 69. | 31 or 36 or 52 or 56 or 60 or 68 |
| 70. | 22 and 69 |

**PubMed (via Medline)**

*1/1/1990-2/20/2016*

"Hepatitis C"[Mesh Terms] OR "hepatitis C" OR "hep c" OR "HCV" OR "hepatitis non a non b"

AND

"Substance Abuse, Intravenous"[MeSH Terms] OR "people who inject drugs" OR "persons who inject drugs" OR PWID OR IDU OR IVDU OR "Substance-Related Disorders"[MeSH Terms] OR "Drug Users"[MeSH Terms] OR "substance use" OR "substance abuse" OR "substance dependence" OR "drug use" OR "drug abuse" OR "drug dependence"

AND

"Mass Screening"[MeSH Terms] OR "Point-of-Care Testing"[MeSH Terms] OR "Hepatitis C Antibodies"[MeSH Terms] OR screening[Title/Abstract] OR testing[Title/Abstract] OR "viral load"[Title/Abstract] OR "Continuity of Patient Care"[MeSH Terms] OR "Delivery of Health Care"[MeSH Terms] OR "Health Services Accessibility"[Mesh Terms] OR "Symptom Assessment"[MeSH Terms] OR "Referral and Consultation"[MeSH Terms] OR assessment[Title/Abstract] OR referral[Title/Abstract] OR "linkage to care"[Title/Abstract] OR "Patient Acceptance of Health Care"[Mesh Terms] OR "treatment willingness"[Title/Abstract] OR "treatment uptake"[Title/Abstract] OR "treatment initiation"[Title/Abstract] OR "treatment adherence"[Title/Abstract] OR "treatment completion"[Title/Abstract] OR "engagement in care"[Title/Abstract] OR "care engagement"[Title/Abstract] OR "treatment engagement"[Title/Abstract] OR "retention in care"[Title/Abstract] OR "care retention"[Title/Abstract] OR "retention in treatment"[Title/Abstract] OR "treatment retention"[Title/Abstract] OR "Treatment Outcome"[MeSH Terms] OR "sustained virologic response"[Title/Abstract] OR "SVR"[Title/Abstract] OR "Recurrence"[Mesh Terms] OR "recurrence"[Title/Abstract] OR "reinfection"[Title/Abstract] OR "re-infection"[Title/Abstract] OR "care continuum"[Title/Abstract] OR "continuum of care"[Title/Abstract] OR "care cascade"[Title/Abstract] OR "model of care"[Title/Abstract] OR "care coordination"[Title/Abstract] OR "coordination of care"[Title/Abstract]
